# Periodicity Pitch Perception

**DOI:** 10.3389/fnins.2020.00486

**Published:** 2020-06-04

**Authors:** Frank Klefenz, Tamas Harczos

**Affiliations:** ^1^Fraunhofer Institute for Digital Media Technology IDMT, Ilmenau, Germany; ^2^Auditory Neuroscience and Optogenetics Laboratory, German Primate Center, Göttingen, Germany; ^3^audifon GmbH & Co. KG, Kölleda, Germany

**Keywords:** periodicity pitch, temporal receptive fields, inter-spike interval tuned microcircuits, first spike latency, periodicity, auditory model

## Abstract

This study presents a computational model to reproduce the biological dynamics of “listening to music.” A biologically plausible model of periodicity pitch detection is proposed and simulated. Periodicity pitch is computed across a range of the auditory spectrum. Periodicity pitch is detected from subsets of activated auditory nerve fibers (ANFs). These activate connected model octopus cells, which trigger model neurons detecting onsets and offsets; thence model interval-tuned neurons are innervated at the right interval times; and finally, a set of common interval-detecting neurons indicate pitch. Octopus cells rhythmically spike with the pitch periodicity of the sound. Batteries of interval-tuned neurons stopwatch-like measure the inter-spike intervals of the octopus cells by coding interval durations as first spike latencies (FSLs). The FSL-triggered spikes synchronously coincide through a monolayer spiking neural network at the corresponding receiver pitch neurons.

## Introduction

Pitches span a scale from lowest to highest pitch. The frequencies of the pitches are determined by adjusting them to an absolute reference pitch (e.g., the concert pitch A4 = 440 Hz) and the chosen temperament. Very seldom the reciprocal interval duration time is annotated for a given frequency. By doing this, it becomes clear that the 25 notes in the mostly played range from C4 to C6 populate an interval time range of about 3 ms only. Periodicity pitch detectors need as prerequisite precise stopwatch-like timers (Buonomano, 2017; Buzsáki and Llinás, 2017; derNederlanden et al., 2018). Our self-developed ANF spike from audio generation program SAM is used as audio front end (Harczos et al., 2013a). We recently extended SAM by model octopus cells innervated by ANFs (Harczos and Klefenz, 2018). These models are shortly summarized for better comprehensibility in see section “Materials and Methods.” Batteries of interval-tuned neurons (ITNs) stopwatch-like measure the inter-spike intervals (ISIs) of assigned octopus cells. An ITN responds to a range of interval durations of a rhythmically spiking octopus cell by coding interval durations as first spike latencies (FSLs) (Aubie et al., 2009, 2012). We model interval-tuned microcircuits by adapting Aubie’s model to be ready for use in the microsecond operating range (Aubie et al., 2012). Aubie’s model is formulated in NEURON with excitatory NMDARs/AMPARs and GABAergic inhibition (Kirst et al., 2017). The parameter search space of the modified model is pruned by various simulation runs led by optimality criteria. ITNs are star-wise connected to short-term pitch neurons in a monolayer spiking neural network (SNN), which processes synchronously arriving spikes from the ITNs.

## Materials and Methods

We like to show a bio-plausible way of F0 estimation as a possible starting point for novel research. As a prerequisite, auditory models of pitch perception have been created, implemented, and discussed (Patterson et al., 2002; Laudanski et al., 2014; Langner, 2015; Stolzenburg, 2015; Ahmad et al., 2016; Joris, 2016; McLachlan, 2016; Barzelay et al., 2017; Friedrichs et al., 2017; Saeedi et al., 2017; Tang et al., 2017; Todd et al., 2017; Harczos and Klefenz, 2018; Oxenham, 2018; Peng et al., 2018).

Neuro-physiologically parameterized auditory models mimic the dynamics of the basilar membrane, the mechano-electrical coupling of inner hair cells to it, and the membrane voltage regulated vesicle rate kinetics into the synaptic cleft between them and the associated auditory nerve fibers (Baumgarte, 1997; Sumner et al., 2002; Yu et al., 2009; Meaud and Grosh, 2012; Harczos et al., 2013a; Zilany et al., 2014; Cerezuela-Escudero et al., 2015; Lee et al., 2015; Ó’Maoiléidigh and Hudspeth, 2015; Saremi et al., 2016; Rudnicki and Hemmert, 2017; Manis and Campagnola, 2018; Saremi and Lyon, 2018; Xu et al., 2018; Liu et al., 2019).

**FIGURE 1:**
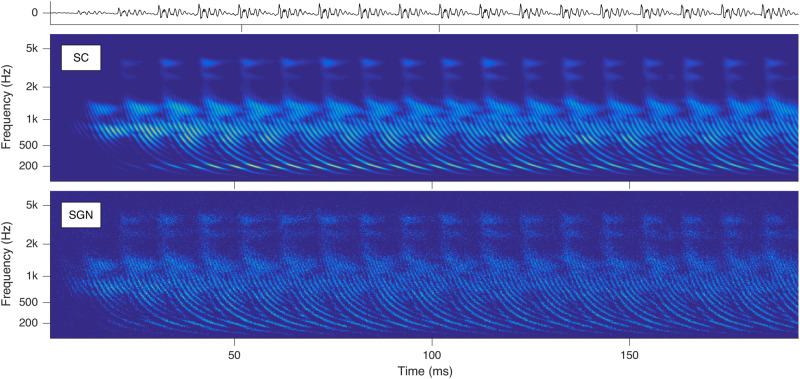
Cochleagrams with quasi-stationary repetitive patterns for a short snippet of the vowel *a:* sung by a male singer at the note of G2. Top: Sound signal waveform. Middle: Probability (ascending from blue over green to yellow) of neurotransmitter substance release into the synaptic cleft (SC) as a function of time and place within the cochlea. Bottom: Action potentials of the spiral ganglion neurons (SGN). Note that the ordinate shows the characteristic frequency of the basilar membrane model at the corresponding cochlear position (Reprint from Harczos and Klefenz, 2018).

Stimulation based on auditory modeling (SAM) – developed at Fraunhofer IDMT as a cochlear implant sound-processing strategy – converts sounds to parallel spike trains along the auditory nerve fibers (ANFs) (Harczos et al., 2013b; Harczos, 2015). With SAM’s auditory model, cochleagrams with characteristic repetitive latency-phase trajectories can be generated as shown in Figure 1.

Stimulation based on auditory modeling has been extended step by step by further modules of the auditory periphery. Octopus cells are topologically arranged in frequency-ordered laminae and locally wired to bundles of ANFs. The wiring patterns’ scheme constitutes their temporal receptive fields (TRFs) (Oertel et al., 2017; Spencer et al., 2018). Octopus cells latency-phase rectify space–time trajectories in their TRFs (Golding and Oertel, 2012; McGinley et al., 2012). Octopus cells rhythmically spike with the pitch periodicity of the sound because they decode repetitively occurring latency-phase trajectories (Harczos and Klefenz, 2018).

**FIGURE 2:**
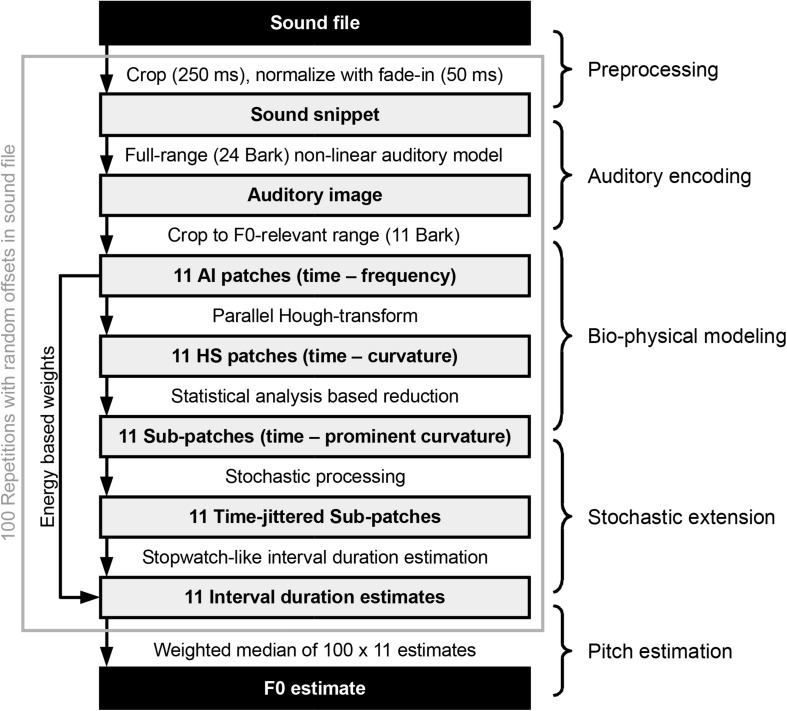
Overview of the processing steps from a single sound file to the pitch estimate (Adapted from Harczos and Klefenz, 2018).

The processing chain employed in this paper can be summarized as shown in Figure 2. A random (uniformly distributed) offset is selected for the specified input sound file. Starting from there, a 250-ms-long snippet is cropped from the file. Then, the sound snippet’s amplitude is normalized to yield around 65 dB SPL in the subsequent auditory model. Next, a 50-ms-long linear fade-in is applied to the snippet, which is then fed to the auditory model introduced above. The output of the auditory encoder, a spectro-temporal representation of sound, is reduced to a pitch-relevant (*F_*mi*__*n*_* = 75 Hz and *F_*ma*__*x*_* = 1500 Hz in the current implementation) 11 Bark frequency range, each of which we address as one of 11 auditory image (AI) patches, the RMS energy of which are stored to be used later as weights for the final F0 estimate. The 11 AI patches are analyzed by an ensemble of dedicated octopus cells. Each octopus cell is tuned for a specific local hyperbolic shape section and is, therefore, part of the distributed Hough-transform execution. This step results in 11 Hough-space (HS) patches, which, based on the maximal variance across the time axes, get reduced to narrower sub-patches. Corresponding to Aubie’s model (as mentioned above and explained in more detail in see section “Interval-Tuned Microcircuits”), we introduce a stochastic processing in the form of Poisson-type jitter added to the timing of the sub-patches. Subsequently, we look for periods using autocorrelation-based interval estimation to yield 11 interval duration estimates, one for each sub-patch. In the present study, the above process is repeated 100 times using 100 different processing offsets within the same input sound file. The aggregated 100 × 11 interval duration estimates along with the 100 × 11 weights (based on the AI patches) are used in the final step to calculate the F0 estimate for the sound file.

For the bio-physical modeling part of the extended SAM front-end, we kindly refer to our previous open access paper (Harczos and Klefenz, 2018), the mathematical notation and symbols of which we continue using throughout the rest of this paper.

### Test Corpora

For testing the presented system, we used three kinds of sounds: pure tones, sung vowels (*a:* and *i:* sung by a female as well as a male singer), and solo instruments (violin, flute, and piano). The latter were taken from the McGill University master samples (MUMS) CDs (Opolko and Wapnick, 1987) and correspond to CD1 Track 6 (violin, bowed), CD2 Track 5 (alto flute), and CD3 Track 3 (9′ Steinway grand piano, plucked). The sung vowel database was created at the Fraunhofer Institute for Digital Media Technology (IDMT) and can be obtained free of charge by contacting the authors.

### Interval-Tuned Microcircuits

Periodicity pitch is derived from joint analysis of octopus inter-spike intervals (ISIs), where the reciprocal of the dominant interval is considered to be the pitch. Octopus ISIs are measured by interval duration metering units, which operate in the range between a shortest interval duration *t_*mi*__*n*_* and a largest interval duration *t_*ma*__*x*_* (Paton and Buonomano, 2018). Interval-tuned neurons (ITNs) have been identified in various species (Hedwig, 2016; Rose, 2018; Yamada et al., 2018). The interval duration metering unit is a stopwatch started by interval onset and stopped by interval offset. The stopwatch is triggered by a first spike of an octopus cell and stopped by the consecutive one, thus metering the time interval between them. The interval measuring unit is effectuated by an IC neuron. We name the IC neuron from here on as the intermittently interval-tuned neuron (ITN).

The stopwatch requires three start/stop control signals to the ITN: onset-evoked excitation, offset-evoked excitation, and onset-evoked inhibition, which is sustained for equally long or longer than the interval duration. The axons of octopus cells trifurcate to excitatory MSO_ON neurons, excitatory MSO_OFF neurons, and inhibitory DNNL_ON neurons, whose outputs, in turn, project to the associated ITNs as common terminals.

**FIGURE 3:**
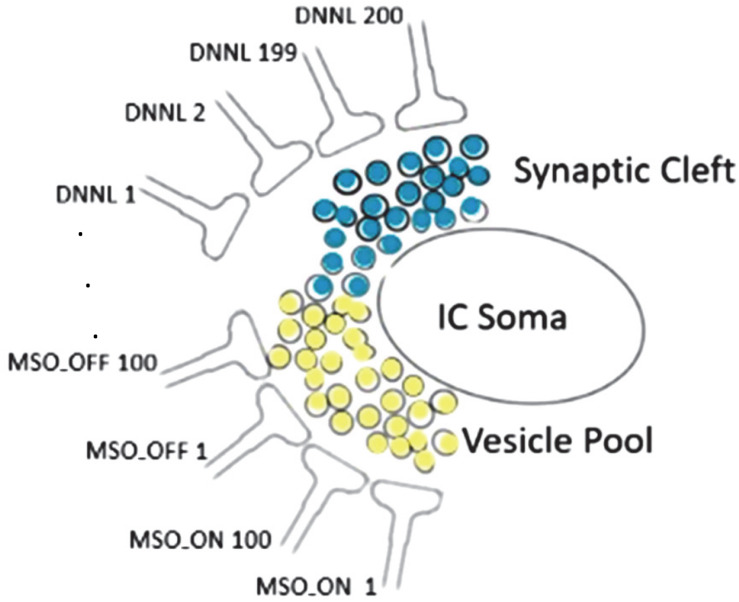
Synaptic cleft neurotransmitter releases (light yellow: glutamate, light blue: GABA) sourced from 100 MSO_ON, 100 MSO_OFF, and 200 DNLLs synapses; each neurotransmitter release from an exocytosed vesicle is indicated by a black surrounding circle; any spillover vesicle tilts the excitatory/inhibitory balance and triggers a spike at the IC soma with high temporal fidelity.

The interval duration registering timer unit has an internal sandglass-like mechanism substituting metaphorically sand particles by neurotransmitter vesicles (Figure 3). Any spillover vesicle tilts the excitatory/inhibitory balance (Gandolfi et al., 2020).

The complex temporal interplay of ON/OFF excitation and ON inhibition makes the timer unit selective for interval durations (Wehr and Zador, 2003; Edwards et al., 2008; Simen et al., 2011; Takizawa et al., 2012; Naud et al., 2015; Majoral et al., 2018; Rajaram et al., 2019). At the start, the IC soma is charged by mini EPSCs by MSO neurons and discharged by IPSCs by DNLL neurons. If the critical total equilibrium of balanced net EPSC and IPSC inputs passes threshold, a highly timely precise IC soma-initiated spike is triggered.

In principle, an onset-evoked excitation temporally coincides with an offset-evoked excitation and produces spikes in an ITN when the onset-evoked excitation has a compensatory latency equal to the interval duration (Simmons and Simmons, 2011). In the presence of inhibition, neither the onset- nor offset-evoked excitations are supra-threshold on their own and cannot evoke spiking in the ITN; however, when the onset- and offset-evoked excitations temporally coincide, the summed excitation can overcome inhibition and evokes spiking in the ITN (Aubie et al., 2009, 2012; George et al., 2011; Buhusi et al., 2016; Akimov et al., 2017; Kopp-Scheinpflug et al., 2018; Baker et al., 2019; Felmy, 2019).

For a computational stopwatch implementation, we take over and adapt Aubie’s timer model (Aubie et al., 2012). The ITN is composed as a single-compartment IC soma with a diameter of 13 μm equipped with glutamate-activated excitatory depolarizing AMPA, NMDA, and inhibitory hyperpolarizing GABA_A_ ion channels. Receptor kinetics is based on the simplified versions of postsynaptic currents from the study by Destexhe et al. (1998). Briefly, presynaptic spikes trigger a 1-ms release of a 1-mM neurotransmitter that activates postsynaptic receptor currents with kinetics specified in Aubie et al. (2012). A spike is triggered at the time step in which the membrane potential of the ITN neuron crosses 0 mV. The rates of neurotransmitter binding α and unbinding β determine the rise and decay kinetics of each postsynaptic receptor conductance *g*_*AMPA*_, *g*_*NMDA*_, and *g_*GABA*__*A*_* (Rowat and Greenwood, 2014). Fitted parameter values for α and β were previously determined from whole-cell current recordings (Destexhe et al., 1998). NMDA receptors exhibited a voltage-dependent Mg^2+^ block characterized by the function *B*(*V*) as defined by Jahr and Stevens (1990). The membrane also contains passive channels that conduct leak current *I*_*leak*_ and channels for fast Hodgkin–Huxley-type sodium *I*_*Na*_ and potassium *I*_*K*_ currents based on the kinetics described by Traub and Miles (1991) and implemented by Destexhe et al. (1996). Voltage dynamics of the model IC cell membrane potential *dV*/*dt* were determined by the following equation:

(1) $$C_{m}\times \left(\frac{d\,V}{d\,t}\right)=I_{l\,e\,a\,k}-I_{N\,a}-I_{K}-I_{A\,M\,P\,A}-I_{N\,M\,D\,A}-I_{G\,A\,B\,A_{A}}$$

where *C*_*m*_ is the membrane capacitance; *I*_*leak*_ the passive membrane leak current; *I*_*Na*_ the sodium channel current; *I*_*K*_ the potassium channel current; and *I*_*AMPA*_, *I*_*NMDA*_, and *I_*GABA*__*A*_* the corresponding receptor-mediated currents.

Presynaptic spikes that activate glutamatergic AMPA and NMDA receptors on the ITN are generated by two single-compartment excitatory neurons: one providing excitation timed relative to a first octopus spike (onset-evoked stimulus, MSO_ON) and the consecutive offset-evoked stimulus (MSO_OFF) (Oertel et al., 2019). Presynaptic neurons were modeled with fast-spiking kinetics such that a 1-ms, 0.1-nA injected current pulse produces exactly one spike in the neuron. IPSPs are modeled with GABA_A_ receptor kinetics. Inhibitory presynaptic inputs to the ITN are generated by a population of single-compartment presynaptic inhibitory neurons with fast-spiking kinetics that activate GABA_A_ receptors on the model ITN. A current of discrete 1-nA square pulse in a simulation time step of 0.05 ms is injected into each inhibitory presynaptic neuron. In Aubie’s model, the inhibitory DNNL neurons randomly fire with a Poisson distribution. This is simulated by injection times following a Poisson distribution with a mean probability of 0.05 events per time step (i.e., on average, each presynaptic neuron received 1 nA of current for 0.05 ms per 20 simulation time steps).

### Pitch Estimation Monolayer SNN

Several octopus cells observe local segments of a common global trajectory in their TRFs. Each global trajectory is, therefore, represented by its unique set of spiking octopus cells. In the narrower mathematical sense, the TRFs are time-shifted relative to an imaginary vertical zero line according to their lateral spatial positions. For a given set, all relative time shifts are set to zero in order to achieve a common synchronization.

**FIGURE 4:**
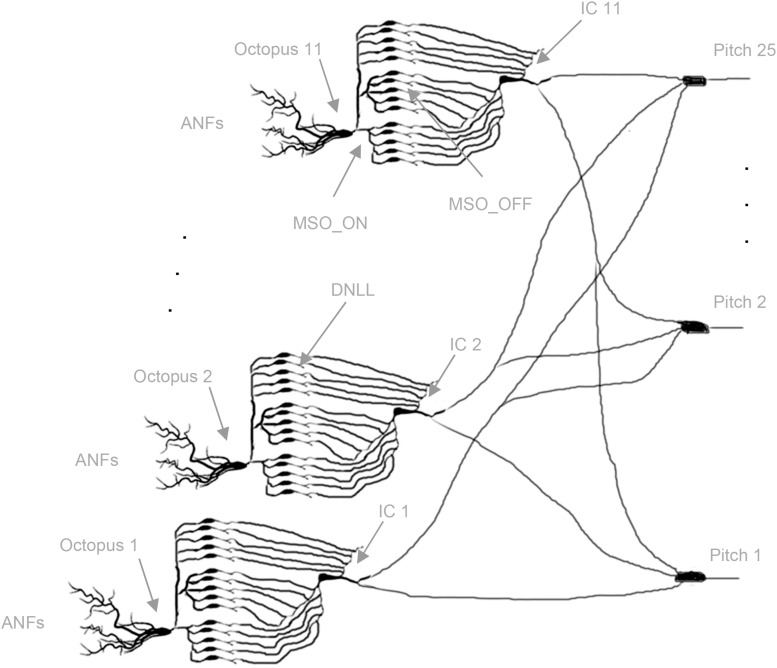
Topology of the neural network. Starting from left, octopus cells receive input from ANFs in their temporal receptive fields (three out of 11 are shown); each dendritic end branch connects to an ANF (not shown). Octopus cells trifurcate to MSO_ON neurons (bottom blocks: five out of 100 shown), MSO_OFF neurons (middle blocks: five out of 100 shown), DNLL neurons (top blocks: five out of 200 shown); MSO_ON, MSO_OFF, and DNLL neurons connect to the bottom, middle, and top dendritic branches of the inferior colliculus (IC) neurons, respectively; IC neurons connect star-like to pitch neurons (three out of 25 are shown) (Cells redrawn from Siveke et al., 2006; Bal and Baydas, 2009; Wallace et al., 2012).

For quasi-stationary tones, global trajectories are repeated almost identically, and almost always the same octopus cells spike. The intra-synchronization for each set assures common arrival times at the ITNs, and in turn, the synchronized FSLs allow a spiking coincidence processing at the pitch neurons (Bagheri et al., 2017). The template matching of global trajectories is transposed to local distributed processing in spiking neural network architectures. A simple monolayer spiking neural network (SNN) with interval-tuned neurons in the input layer, star-wise connected to pitch neurons in the output layer, is constructed (Figure 4; Calixto et al., 2012; Bidelman, 2013; Baumann et al., 2015; Ranjan et al., 2019). Due to the star connectivity, ITNs can contribute to all pitch decisions, and the pitch neurons can collect votes from all ITNs. Each ITN contributes at a specific interval duration FSL time *t* with a spike, which is weighted by its actual synaptic connection strengths to pitch neurons. Each pitch neuron synchronously receives spikes at the same FSL time *t* for a set of ITNs of the same interval duration. The sum of the activated synaptic weights at isochronous FSL time *t* determines if a pitch neuron reaches threshold and, in turn, spikes (poly-pitch mode).

If only the dominant pitch is to be determined, a *softmax* operation is applied. The standard SNN is replicated *n* times, and each SNN is trained individually for template matching for each global trajectory. Mono and poly pitches can be learned by adjusting the synaptic weights, but learning is outside the scope of this article and is deferred for a subsequent article.

### Implementation Details

The auditory encoder as well as the simulation of the bio-physical model of the pitch estimation has been implemented on a PC platform in an interplay of parts implemented in C, C++, MATLAB, NEURON, and Python languages. For evaluation and data visualization, we used MATLAB R2019a from MathWorks and Microsoft Excel 2010. The network models used in the present study were obtained from ModelDB “Duration-tuned neurons from the inferior colliculus of vertebrates,” accession number 144511 (Aubie et al., 2012). We used NEURON version 7.7 (McDougal et al., 2017) and Python Anaconda 3 (both 64-bit versions) on a Dell Optiplex 7010 under Microsoft Windows 10. NEURON simulations were run with a time step resolution of 0.05 ms.

## Results

### Optimality Criterion

Aubie’s model has a lot of adjustable parameters. To adapt the model to interval duration estimation, the first criterion is to define the operation range by choosing adequate parameter settings that show FSL behavior in response to applied interval durations. The second criterion is to change the original parameter space as little as necessary. The third criterion is optimality by minimization of the mean of FSL standard deviations of a simulation run with pre-given parameter set. To reliably distinguish semitones at the 95% confidence level (±2σ), the condition {2σ_tone+__1_ + 2σ_tone_ < |*FSL_*tone+*__1_*–*FSL_*ton*__*e*_*|} must hold for the two adjacent halves of neighboring distributions.

### Parameter Search Space

Aubie’s model is species-specific, and models for bat, rat, mouse, and anuran are given. As bats rely on hearing, our initial guess was to adopt bat mode, and it turned out to be the best one for the task. Bat mode is defined in module *C_BAT_JUN2.* Various coincidence mechanisms are proposed and evaluated in Aubie’s model, for instance, anti-coincidence and excitatory onset/offset with inhibitory onset. We found that the coincidence mechanism as defined in *network.DTN_Coincidence* works best by minimizing overall mean FSLs in conjunction with *C_BAT_JUN2.*

Starting initially with the mouse model, we ran many simulations with many different parameter settings, and we realized in frustration that we never met the optimality criterion as standard deviations were always too high for the original model. Switching to bat mode helped a little, but still, standard deviations were too high. We decided to systematically search the parameter space by continuously varying a single parameter and clamping it if we found a local minimum. With this fixed parameter, we iterated the simulation and fixed the next parameter and so on.

The operation range in which FSL is a linear function of interval duration could be easily found, and the optimum values are given by *gmax*_*AMPA*_ = 0.006, *gmax*_*NMDA*_ = 0.035, *gmax_*GABA*__*A*_* = 0.001 (see Aubie et al., 2012 for discussion).

The variations of soma time constants t of ITNs and presynaptic neurons had little effect on the standard deviation criterion. Local minima have been reached by setting excitatory MSO_ON neuron soma t to 1 ms, DNLL neuron soma t to 1 ms, and ITN soma t to 5 ms.

We tried to identify those parameters that have a big impact on the results. We realized that the limiting factor of precision is the stochastic process with the Poisson distributed jitter term of varied injection times. To dampen the jitter noise, we changed the model by setting the number of inhibitory neurons to *numDNLL* = 200, excitatory ON neurons to *numMSO_ON* = 100, and excitatory OFF neurons to *numMSO_OFF* = 100. The random jitter of IC soma spiking is attenuated by the high number of 200 DNNLs. The DNLLs fill the vesicle pool (Figure 3). As soon as the vesicle pool reaches subthreshold, the next spillover vesicle excites a spike initiating from the IC soma. The IC soma spike time is very precise as it doesn’t matter which individual DNNL neuron released the spillover vesicle.

### Estimation of Interval Durations

Interval duration times are annotated for semitones and frequencies over two octaves from C4 to C6, in which most melodies are notated (Table 1).

**TABLE 1:** Annotated time interval durations referring to a 12-tone equal temperament relative to A4 (440 Hz).

**Tone**	**Frequency (Hz)**	**Interval (ms)**
C6	1046.5	0.96
B5	987.77	1.01
Bb5	932.33	1.07
A5	880	1.14
Ab5	830.61	1.2
G5	783.99	1.28
Gb5	739.99	1.35
F5	698.46	1.43
E5	659.26	1.52
Eb5	622.25	1.61
D5	587.33	1.7
Db5	554.37	1.8
C5	523.25	1.91
B4	493.88	2.02
Bb4	466.16	2.15
A4	440	2.27
Ab4	415.33	2.41
G4	392	2.55
Gb4	369.99	2.7
F4	349.23	2.86
E4	329.63	3.03
Eb4	311.13	3.21
D4	293.66	3.41
Db4	277.18	3.61
C4	261.63	3.82

The time difference from tone C4 (261.63 Hz) to tone C6 (1046.5 Hz) is 2.86 ms. Twenty-five semitone intervals are allocated within this time span. Due to the reciprocal ratio between interval time and interval frequency, the tone intervals aggregate more densely at short tone intervals and distribute more loosely at longer tone intervals.

These 25 tones are applied to Aubie’s model as the ultimate test of its robustness and reliability to distinguish tone interval durations. In order to mimic the stochastic behavior of neurons, each interval duration trial is repeated 20 times with a randomly varying current injection time (Fisch et al., 2012). The random injection time follows a Poisson distribution effectuated by NEURON pseudo-random generator *Mcell4*. For each interval duration, mean FSL time and standard deviation over 20 trials are computed. This amounts, with 25 note interval times and 20 repetition trials each, to 500 simulations per run.

Only minor task-specific changes have to be made to the original model. Most parameters of the model, explicitly the AMPA, NMDA, and GABA_A_ receptor kinetics and the sodium, potassium, and passive leakage channel kinetics as well as the channel kinetics of the presynaptic excitatory and inhibitory model neurons, remain unchanged. All necessary parameter changes are explicitly indicated so that results are externally reproducible by third parties.

### First Spike Latency (FSL)

The interplay of onset-/offset-evoked excitations and onset inhibitions triggers response stimulus onset times in ITNs relative to onset-evoked excitation trigger time. The difference of response stimulus onset time and onset-evoked excitation time is defined as first spike latency (FSL). FSLs systematically increase for ascending tone-interval durations. FSL starts with *FSL*_*mindur*_ for the best matching interval and ends with *FSL*_*maxdur*_ for the largest deviant interval duration. FSL depends on the species and the chosen coincidence mechanism.

**FIGURE 5:**
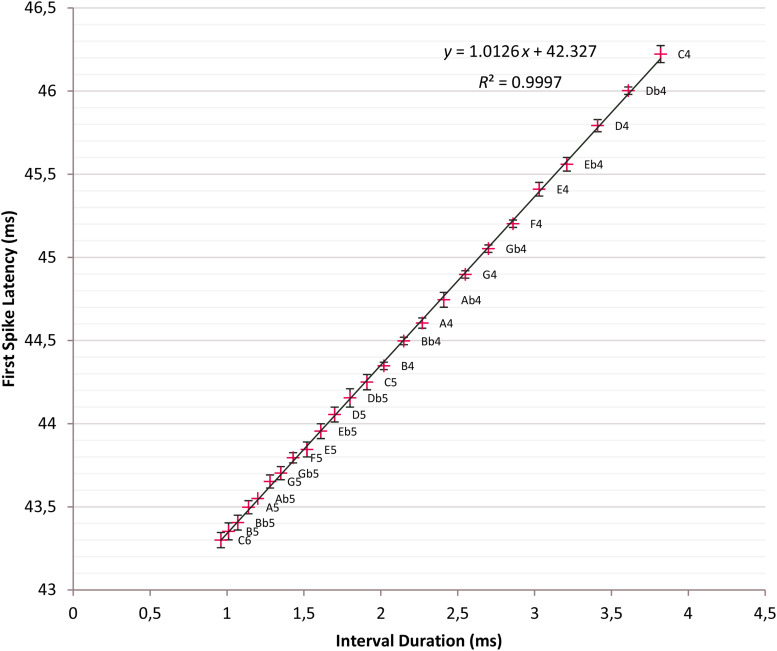
Time intervals versus first spike latencies (FSLs). Crosses: Tone intervals and corresponding FSLs; t = 5 ms; linear regression line fitted: *y* = 1.0126*x* + 42.327, *R*^2^ = 0.9997; error bars ±2σ; 95% confidence interval.

Other influential parameters on response stimulus onset time and, hence, FSL are the membrane time constant t of the soma of the ITN, which is the product of membrane resistance *r*_*m*_ and membrane capacitance *c*_*m*_; others are receptor conductances *g* of AMPA, NMDA, and GABA_A_ receptors (see Aubie et al., 2012 for discussion). For the best parameter setting, we determined C6 produces the minimal mean first spike latency *FSL*_*mindur*_ of 43.3 ms and C4 the maximal mean first spike latency *FSL*_*maxdur*_ of 46.22 ms. The FSLs over two octaves C4 to C6 with 25 semitone intervals fall between *FSL*_*mindur*_ and *FSL*_*maxdur*_. The data points are plotted with ±2 σ error bars in a common diagram and a regression line is fitted (Figure 5).

First spike latencies are a linear function of the tone interval with the regression line given as *y* = 1.0126*x* + 42.327; *R*^2^ = 0.9997. Interval durations and FSLs are nearly identical because of the slope 1.0126 of the regression line. This setting has a drastic impact and minimizes the mean standard deviation over C4 to C6 to 18.11 μs, which is an indicator for the high precision of the timers. From C4 until F5, except a single slight overlap {Db5, D5}, there is no overlap of the ±2 σ error bars so that tones are distinguishable with high fidelity at the 95% confidence level.

### Stochastic Term Modeling

The adapted Aubie’s model responds with a mean FSL *SD* derived from all intervals of 18.11 μs. To circumvent the CPU’s time-consuming interval duration computation in NEURON, for every ISI we replace Aubie’s model by formulating an equivalent stochastic computation input/output function with a Poisson distribution of ±20 μs and apply it to the test corpora. We take audio snippets with a length of 100 consecutive octopus spike intervals for a selected patch. For each interval, we compute a mean F0 for each patch. The computation of the weight of a patch is the same as in our previous article (Harczos and Klefenz, 2018).

### Individual Sound Categories

**FIGURE 6:**
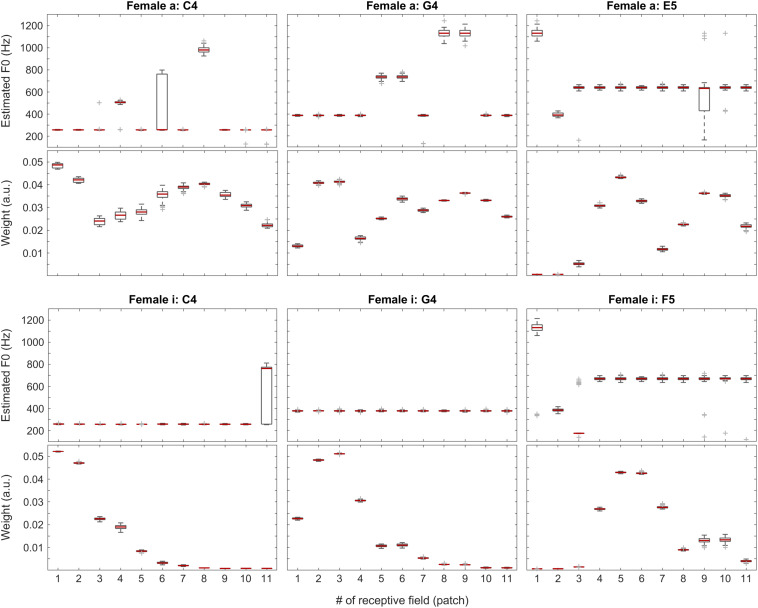
F0 estimates (first and third rows) and weights (second and fourth rows) for all patches over all 100 iterations for the vowels *a:* (top two rows) and *i:* (bottom two rows) sung by a female singer.

**FIGURE 7:**
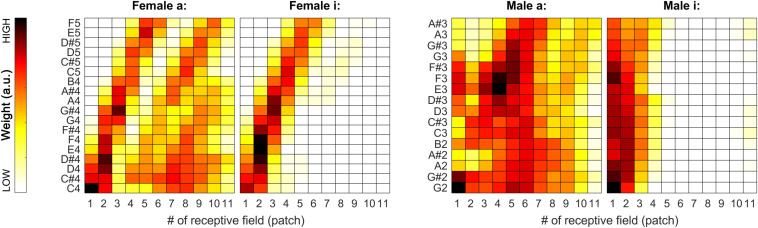
Median of the weights (over all 100 iterations) for the complete tested pitch range for the sung vowel recordings. From left to right: *a:* and *i:* by female singer, then *a:* and *i:* by male singer.

**FIGURE 8:**
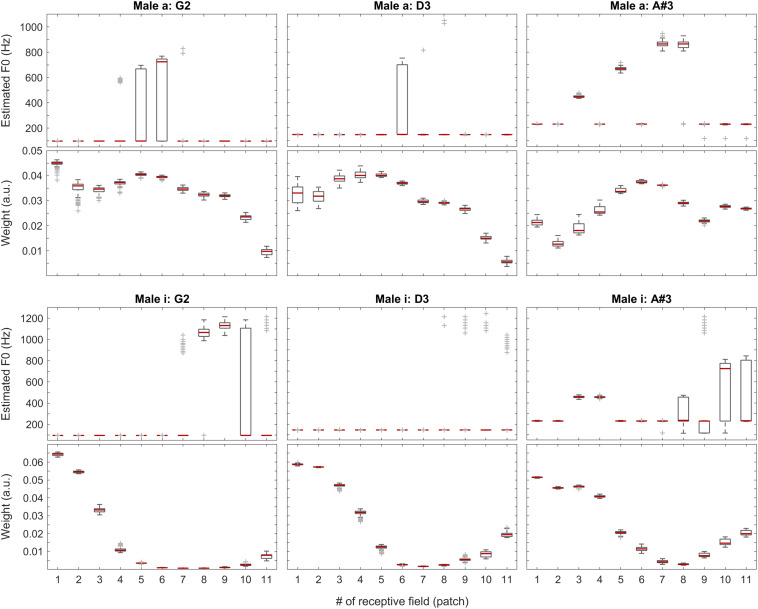
F0 estimates (first and third rows) and weights (second and fourth rows) for all patches over all 100 iterations for the vowels *a:* (top two rows) and *i:* (bottom two rows) sung by a male singer.

**FIGURE 9:**
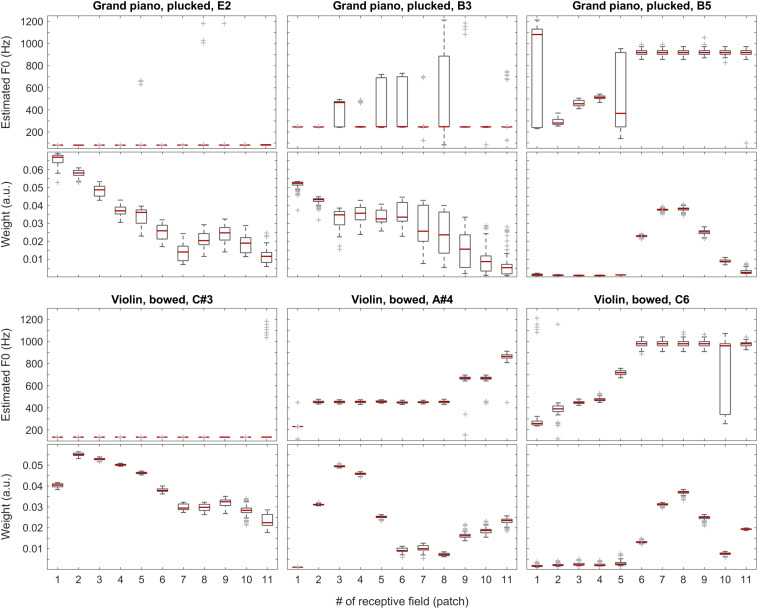
F0 estimates (first and third rows) and weights (second and fourth rows) for all patches over all 100 iterations for the grand piano (top two rows) and violin (bottom two rows) recordings.

**FIGURE 10:**
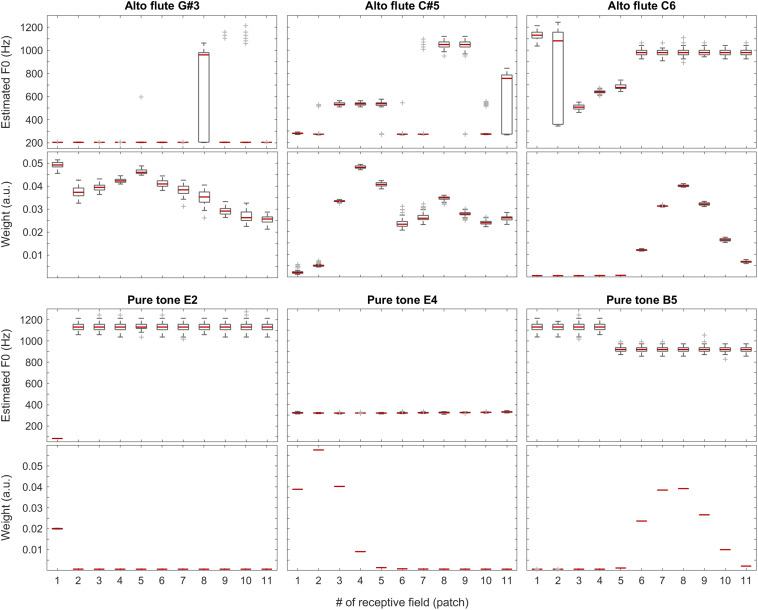
F0 estimates (first and third rows) and weights (second and fourth rows) for all patches over all 100 iterations for the alto flute recordings (top two rows) and pure tones (bottom two rows).

**FIGURE 11:**
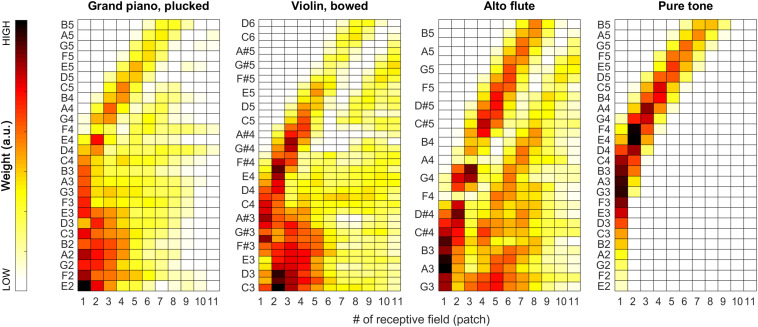
Median of the weights (over all 100 iterations) for the complete tested pitch range for the grand piano (leftmost plot), the violin (second plot), and the alto flute recordings (third plot), and for pure tones (rightmost plot).

To understand the inner workings as well as the strengths and weaknesses of our compound model, we tracked and visualized both the weights and the F0 estimates for each patch in every one of the 100 iterations for all the test files. Without claiming completeness, below in Figures 6–11, we present a few examples for each tested category along with an overview of the weights for all test sounds (median over all iterations). In these figures, the central marks of the boxes (colored in red) indicate the medians. The 25th and 75th percentiles are represented by the bottom and the top edges of the boxes, respectively. The whiskers extend to the extreme data points, which are not yet considered outliers. Outliers are at least 1.5 interquartile ranges away from either end of the box.

In Figure 6, positions of the maximum weights seem to follow F0 nicely, whereas the profiles of the weights also correlate well with the formants of the given vowels. When looking at the single-patch F0 estimates, on one hand, we find a few instances deviating from the correct F0 estimate, which, on the other hand, is represented by the majority of the receptive fields. When we attach the weights to the F0 estimates, i.e., when we calculate the Edgeworth type weighted median as the aggregate fundamental frequency estimate for the given sound snippet (not shown here), we get the correct F0 estimate in all the above cases.

During our tests, weights proved to be very stable (i.e., have low spread around their median) over the iterations, so we decided to also visualize the median weights alone for all tested pitches for all sound categories. In Figures 7, 11, pitch increases from bottom to top. The heat-map colors ranging from white over yellow and red to black correspond to increasing weights. Because the weight units are arbitrary, plots are normalized separately and do not necessarily cover the same range of weights.

As apparent from Figure 7, the weights can provide a beneficial extension to the single-patch F0 estimates by prioritizing those belonging to high-energy auditory image patches. This applies particularly to the vowels sung by the female singer (see left two plots in Figure 7), and the resolution of formants was far less efficient for the much lower pitched male singer (see right two plots in Figure 7). For details, please also evaluate Figure 8.

With the instruments piano and violin, we observed similar performance of the system: although the F0 of low-pitched sounds are estimated accurately in all receptive fields, with higher-pitched notes, the extent of ambiguity and the number of mispredictions increase as shown in Figure 9, below. Nevertheless, when we attach the weights (see also the first two plots in Figure 11) to the single-patch F0 estimates (Edgeworth type weighted median as discussed above), the combined F0 estimates are correct in all cases.

The sound of the alto flute instrument is characterized by its rich, mellow tone, at least in the lower portion of its range, which is also represented by the weights shown in the second row of Figure 10 and the third plot in Figure 11. Although, with increasing pitch, the single-patch F0 estimates diverge more, the weight profiles get peakier and increasingly localized at the same time. Thus, the combined F0 estimates tend to remain accurate.

The situation is similar but more striking with pure tones for which the data is shown in the bottom half of Figure 10 and in the last plot of Figure 11. With a pure tone (sine wave), there is no harmonic structure in the spectrum, just a well-defined peak, which leaves many weights (deduced from the activity specific to individual frequency bands within the tonotopically organized auditory system) near a value of zero. In the corresponding receptive fields, the single-patch F0 estimates are often not even in the right ballpark; however, they also do not have much impact on the combined F0 estimates due to their low associated weights.

**FIGURE 12:**
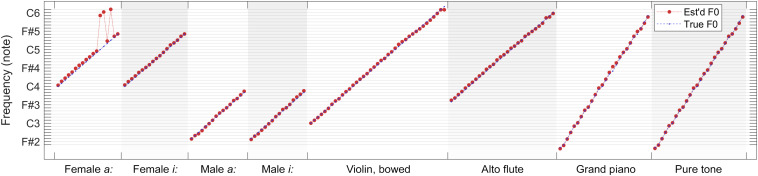
Comparison of true fundamental frequencies versus combined F0 estimates (weighted median over 100 iterations) for each tested note within each sound category.

As a summary, in Figure 12, we present a comparison of true fundamental frequencies versus combined F0 estimates (weighted median over 100 iterations) for each tested note within each sound category. It is apparent in the overview that F0 estimates follow true fundamental frequencies remarkably well for all but four test files (female sung vowel *a:* at C5, C#5, and D#5, and violin at D6). In all other cases, the errors are moderate enough for a subsequent quantizer to predict the played musical note correctly.

## Discussion

We see our main contribution in adapting Aubie’s model to tone interval-duration estimation. Spatiotemporal trajectories of ANF spike trains are latency-phase rectified by dendritic trees of octopus cells via modeling the execution of mathematical Hough-transforms as, for instance, discussed too for visual processing in the LGN and V1 (Barlow, 1986; Blasdel, 1992; Akima et al., 2017; Alam et al., 2017a). Batteries of interval-tuned neurons estimate tone interval durations of successively spiking octopus cells and a monolayer SNN recombines all ITN votes of different layers for short-term pitch estimation (McGinley et al., 2012; Spencer et al., 2012; Wang and Liu, 2013). The model leaves ample space for discussion. Is the stochastic term reasonable or has the model to be refined and reformulated as it works unambiguously only up to the limit tone F5? Are there neurophysiological correlates, which justify the number of excitatory and inhibitory neurons used to fulfill the optimality criteria constraints? Can unprecise mean FSL short-term votes lead to a resolution of tones beyond F5 by accumulating the votes of many ITNs and integrating the short-term votes over the whole tone duration period? We aim to seek answers to these questions in follow-up studies.

The system can be extended to estimate poly-pitches. In this case, the general *softmax* operation has to be substituted by a poly-pitch analysis method as in Elvander et al. (2016). A higher auditory authority needs to reconcile the votes from all interval neurons by sorting out false pitch votes and accepting the right ones (Tabas et al., 2019). In such a system, decisions about wrong and right votes are based on empirical knowledge the system would need to have gathered previously, which implies the need for some kind of learning components (Alam et al., 2017b).

Aubie’s model is formulated in NEURON; hence, a targeted neuromorphic hardware needs to support the portability of NEURON code by an application programming interface. Benchmarking of neuromorphic hardware systems helps to define standardized criteria of code mapping, execution, and measuring performance (Ostrau et al., 2020). A few neuromorphic hardware resources are available (Thakur et al., 2018). A hardware emulation is feasible if the hardware specifications support the model and reproduce the results in the optimal case one by one. High-fidelity reproduction of ionic channel rate kinetics with optimal solid state neurons is recently reported (Abu-Hassan et al., 2019). Many neuromorphic systems lack either the AMPA and/or the GABA channels; thus, the model can be implemented only partly (Benjamin et al., 2014; Furber et al., 2014; Merolla et al., 2014; Yang et al., 2015, 2018, 2020). A promising candidate is Spikey with its PyNN application programming interface, which allows execution of NEST and NEURON code (Pfeil et al., 2013). NeuroSoc seems to be the ideal candidate because NMDA, AMPA, and GABA channel kinetics are supported (Mayr et al., 2015; Keren et al., 2019). An accelerated analog neuromorphic hardware system emulating NMDA- and calcium-based non-linear dendrites is a promising candidate too (Schemmel et al., 2017). To realize the large number of MSO, DNLL, IC neurons in hardware is misleading as the realized timers can be easily substituted in hardware with precise clockwork mechanisms. An elegant way to implement the model seems to be a hardware-friendly unsupervised memristive neural network with a weight-sharing mechanism (Tang et al., 2019). Start and stop switches control the settings of time intervals that are collectively memorized in a common stack of memristor cells.

## Conclusion

Stimulation based on auditory modeling’s auditory model extended by octopus ensembles and batteries of interval-tuned microcircuits reliably extracts periodicity pitch until the limit tone F5. Multi-vesicular releases triggered by many MSO_ON, MSO_OFF, and DNLL_ON neurons allow a time-accurate collective filling of the vesicle pool at the soma of an ITN. Despite the Poisson-distributed stochastic firing times of the pre-neurons, the vesicular spillover fine-dosed by the threshold setting leads to an ultra-precise stopwatch behavior. In the given working range, the system effectively levers out the pitch dichotomy of place and periodicity.

## Data Availability Statement

The raw data supporting the conclusions of this article will be made available by the authors, without undue reservation.

## Author Contributions

FK and TH designed and formulated the model, implemented and tested the model, wrote the manuscript, and edited the manuscript.

## Conflict of Interest

The authors declare that the research was conducted in the absence of any commercial or financial relationships that could be construed as a potential conflict of interest.
