# Erratum: Periodicity Pitch Perception

**DOI:** 10.3389/fnins.2020.00833

**Published:** 2020-08-04

**Authors:** Frontiers Production Office

**Affiliations:** Frontiers Media SA, Lausanne, Switzerland

**Keywords:** periodicity pitch, temporal receptive fields, inter-spike interval tuned microcircuits, first spike latency, periodicity, auditory model

Due to a production error, microseconds (μs) were erroneously changed to milliseconds (ms). A correction has been made to the ***Results***, subsection ***First Spike Latency (FSL)***, paragraph three:

“First spike latencies are a linear function of the tone interval with the regression line given as *y* = 1.0126*x* + 42.327; *R*^2^ = 0.9997. Interval durations and FSLs are nearly identical because of the slope 1.0126 of the regression line. This setting has a drastic impact and minimizes the mean standard deviation over C4 to C6 to 18.11 μs, which is an indicator for the high precision of the timers. From C4 until F5, except a single slight overlap {Db5, D5}, there is no overlap of the ±2 σ error bars so that tones are distinguishable with high fidelity at the 95% confidence level.”

Corrections have also been made to the ***Results***, subsection ***Stochastic Term Modeling***:

“The adapted Aubie's model responds with a mean FSL SD derived from all intervals of 18.11 μs. To circumvent the CPU's time-consuming interval duration computation in NEURON, for every ISI we replace Aubie's model by formulating an equivalent stochastic computation input/output function with a Poisson distribution of ±20 μs and apply it to the test corpora. We take audio snippets with a length of 100 consecutive octopus spike intervals for a selected patch. For each interval, we compute a mean F0 for each patch. The computation of the weight of a patch is the same as in our previous article (Harczos and Klefenz, 2018).”

The publisher apologizes for this mistake. The original article has been updated.
